# Supplementary material for: Exposing telomere length’s impact on malnutrition risk among older adults residing in the community: Insights from cross-sectional data analysis
Source: PLoS One. 2024 Nov 5;19(11):e0308612. doi: 10.1371/journal.pone.0308612 (PMC11537379; doi:10.1371/journal.pone.0308612)
Supplement: Attachment — Submitted filename: Response to Reviewers.docx [file pone.0308612.s001.docx]

**Response to reviewer’s:**

**Reviewer’s requests:**

1. Statistical Methods:

* Provide more detail on the rationale for choosing specific statistical tests in the methods section.

**Response: Thank you very much. The information related to the specific statistical tests is now inserted on the methods section point 4 (Statistical analysis) first paragraph**

* Indicate the software used for the statistical analysis, specifically for the regression model.

**Response: Thank you very much. The information related to the regression model’s used software is now inserted on the methods section point 4 (Statistical analysis) last paragraphs.**

2. Practical Applications:

* Include more specific examples of how the findings can be applied in clinical settings to prevent and treat malnutrition in older adults.

**Response: Thank you very much for your advice. We have reformulated the point 6.3 Practical applications.**

3. Additional Analysis:

* Incorporate the model evaluation parameters to provide a more comprehensive evaluation of the model's fit.
* Additionally, if deemed interesting, adding ROC Curve and AUC to provide information on the discriminative power of the model could be valuable.

**Response: Thank you for your advice. We have inserted the following text in results section:** In Figure 2, AUROC represents the quality of the final model, indicating that the significant characteristics were able to explain 71% of the risk of malnutrition.

**We also have inserted figure 2 (**Area under ROC curve representing the fit of final model of logistic regression)**before table 4**

**We also inserted a new note in table 4:** Hosmer–Lemeshow test: p=0.344

4. Add Data Availability Statement:
   * Include a data availability statement as per PLOS ONE's requirements. Describe where the data supporting the findings can be accessed, either as part of the manuscript, its supporting information, or deposited in a public repository. Specify any restrictions on data sharing, if applicable.

**Response: Thank you very much. We have inserted Data Statement information: data will be available if requested to the corresponding author:**

**We would also like to emphasize that we have taken the freedom to replace the term "elderly" with the word "older people" throughout the manuscript to respect the new guidelines of the American Medical Association, the American Psychological Association, Associated Press and the Gerontological Society of America with regard to the elimination of ageist language and to reduce negative stereotypes about older people.** [**https://publichealth.wustl.edu/age-inclusive-language-are-you-using-it-in-your-writing-and-everyday-speech/#:~:text=Terms%20like%20seniors%2C%20elderly%2C%20the,the%20older%20population%20are%20preferred**](https://publichealth.wustl.edu/age-inclusive-language-are-you-using-it-in-your-writing-and-everyday-speech/#:~:text=Terms%20like%20seniors%2C%20elderly%2C%20the,the%20older%20population%20are%20preferred)**.**
